# Trigeminal somatosensation in the temporomandibular joint and associated disorders

**DOI:** 10.3389/fpain.2024.1374929

**Published:** 2024-05-09

**Authors:** Sienna K. Perry, Joshua J. Emrick

**Affiliations:** Department of Biologic and Materials Sciences & Prosthodontics, School of Dentistry, University of Michigan, Ann Arbor, MI, United States

**Keywords:** orofacial innervation, TMJ, TMD, trigeminal, somatosensation, chronic pain, orofacial pain, nociceptors

## Abstract

The temporomandibular joint (TMJ) consists of bone, cartilage, ligaments, and associated masticatory muscles and tendons that coordinate to enable mastication in mammals. The TMJ is innervated by the trigeminal nerve (CNV), containing axons of motor and somatosensory neurons. Somatosensation includes touch, temperature, proprioception, and pain that enables mammals to recognize and react to stimuli for survival. The somatosensory innervation of the TMJ remains poorly defined. Disorders of the TMJ (TMD) are of diverse etiology and presentation. Some known symptoms associated with TMD include facial, shoulder, or neck pain, jaw popping or clicking, headaches, toothaches, and tinnitus. Acute or chronic pain in TMD stems from the activation of somatosensory nociceptors. Treatment of TMD may involve over- the-counter and prescription medication, nonsurgical treatments, and surgical treatments. In many cases, treatment achieves only a temporary relief of symptoms including pain. We suggest that defining the sensory innervation of the temporomandibular joint and its associated tissues with a specific focus on the contribution of peripheral innervation to the development of chronic pain could provide insights into the origins of joint pain and facilitate the development of improved analgesics and treatments for TMD.

## The anatomy of the temporomandibular joint

**Figure 1:**
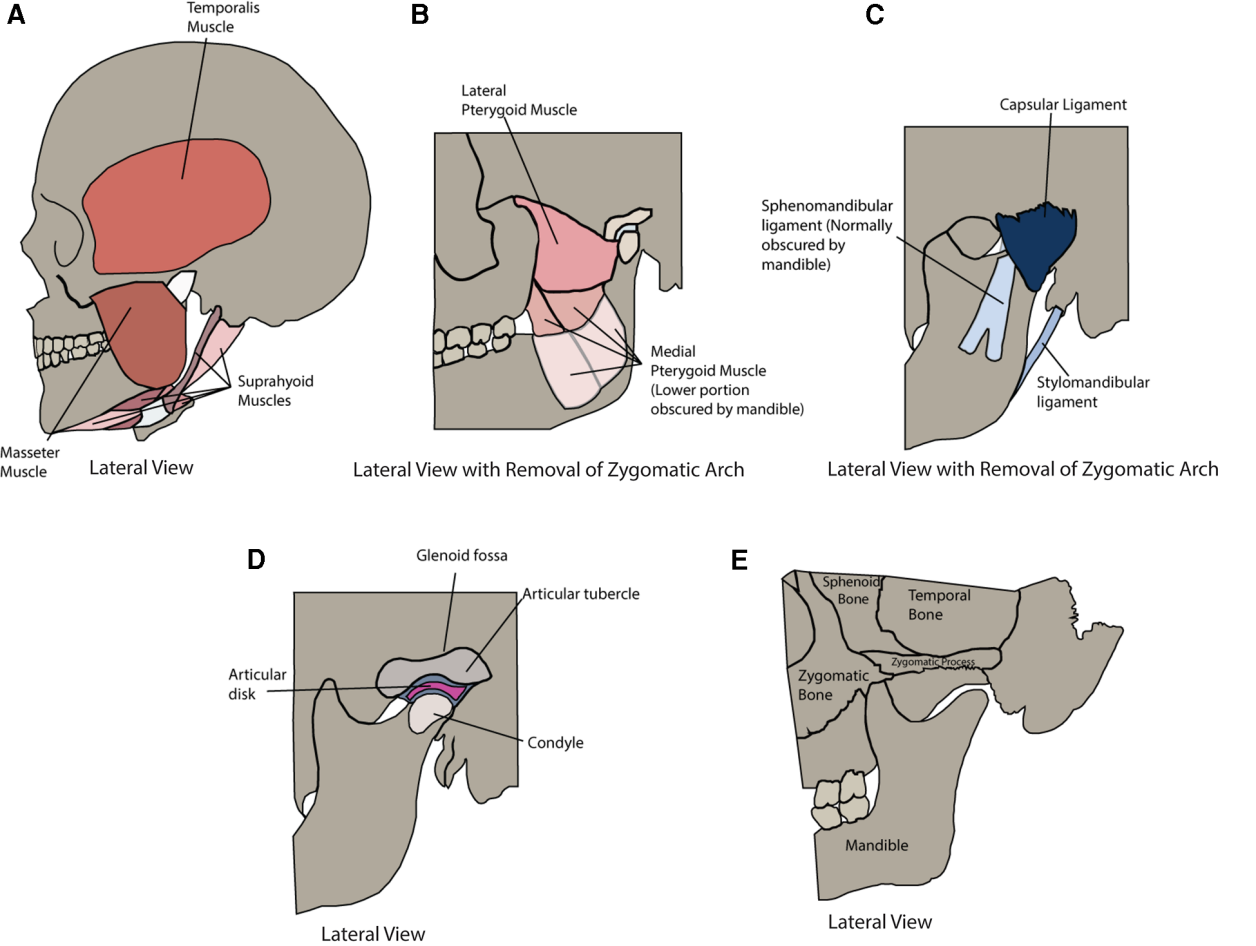
Anatomy of the temporomandibular joint and muscles of mastication. (**A**) Lateral view of the following muscles of mastication: Temporalis Muscle, Masseter Muscle, and Suprahyoid Muscles. (**B**) Lateral view with the removal of the zygomatic arch of the following muscles of mastication: Lateral Pterygoid Muscle and Medial Pterygoid Muscle. (**C**) Lateral view with removal of zygomatic arch of the following ligaments: Sphenomandibular Ligament, Capsular Ligament, and Stylomandibular Ligament. (**D**) Lateral view with removal of zygomatic arch of the Glenoid Fossa, Articular Tubercle, Articular Disk, and Condyle. (**E**) Lateral view of the following bony structures: Sphenoid Bone, Temporal Bone, Zygomatic Process, Zygomatic Bone, and Mandible.

The temporomandibular joint (TMJ) in mammals allows the movements of the mandible required for mastication, feeding, swallowing, breathing, and communication (1). The function of the TMJ relies on the combination of its components and complexity. Figure 1 and Table 1 provide a succinct summary of the anatomical structures associated with the TMJ as well as their functions. Overall, the TMJ comprises a complex assortment of mineralized and soft tissues providing the opportunity to understand how diverse components interact. Notably, the proximity of the types of cartilage within the TMJ is especially attractive as it enables direct comparison of similar but distinct compositions. The TMJ is ginglymoarthrodial, featuring both hinging and gliding movements; bicondylar, permitting simultaneous bilateral movements of the mandible; and synovial with an inner capsule lined by a membranous synovium that produces synovial fluid for lubrication and nutrition of the cartilage and TMJ tissues (2). For these reasons the TMJ may represent a model for understanding the shared elements with other joints as well as its unique features that enable specialized functions.

**Table 1:** Anatomy and function of the temporomandibular joint and muscles of mastication.

	Anatomical structure	Function
Bones	Mandible	Largest, and one of few mobile bones in the skull; its condylar process contacts the glenoid fossa
	Temporal bone	Glenoid fossa of temporal bone contacts the mandibular condyle
Cartilages	Articular disk	Fibrocartilage disk that ensures the condyle does not directly contact the temporal bone; provides mechanical elasticity and contributes to mandibular movement
Ligaments	Capsular ligament	Anchors the articular disk to the condylar process of the mandible
	Temporomandibular ligament	Restrains mandibular movement and prevents compressive forces
	Sphenomandibular ligament	Accessory ligaments that protect joint from excessive condylar translation
	Stylomandibular ligament	Accessory ligament that prevents excessive mandible protrusion
	Pterygomandibular ligament	Accessory ligament that prevents excessive mandible movement
	Malleomandibular ligament	Accessory ligament that protects synovial membrane from surrounding tension
Muscles	Lateral pterygoid	Contraction contributes to depression, protrusion, contralateral lateral deviation of the mandible
	Medial pterygoid	Contraction contributes to elevation, protrusion, and contralateral lateral deviation of the mandible
	Suprahyoid (geniohyoid, mylohyoid, and digastric muscles)	Contraction contributes to depression, and ipsilateral lateral deviation of the mandible
	Temporalis	Contraction contributes to elevation, retrusion, and ipsilateral lateral deviation of the mandible
	Masseter	Contraction contributes to elevation of the mandible

The mammalian nervous system is a network of cells (neurons and supporting glia) that are responsible for collecting information about the environment, integrating inputs, and producing appropriate behavioral responses. It is broadly divided into the central and peripheral nervous systems (CNS and PNS, respectively). The PNS connects the body to the CNS and comprises multiple components: (1) the autonomic nervous system, which includes the sympathetic and parasympathetic neurons that regulate bodily functions without conscious input; (2) the sensory nervous system that relay inputs about the environment from the tissues and organs; and (3) the somatic nervous system that triggers contraction of skeletal muscles (3). All PNS components innervate the structures of the TMJ. While autonomic innervation is essential for modulating the associated vasculature and blood flow of the TMJ (4), this section's overview of TMJ innervation will primarily focus on sensory and somatic divisions, as these are the components that are relevant for pain that arises from the structures, as well as initiating and coordinating movements of the TMJ.

The fifth cranial nerve (CNV) carries axons of sensory and somatic innervation to the TMJ. There are three subdivisions of CNV: the ophthalmic (V1), maxillary (V2), and mandibular (V3) nerves (5) each having corresponding divisions of the trigeminal ganglion (TG). The mandibular nerve is the largest of the three (6) and innervates the cheeks, lips, teeth, and gingiva overlying and within the mandible (5). The mandibular nerve features nine branches, but only three innervate aspects of the TMJ: the masseteric, deep temporal, and auriculotemporal nerves. The masseteric nerve innervates deep aspects of the masseter, the deep temporal nerve innervates the temporalis and masseter (7), and the auriculotemporal nerve innervates the joint capsule and the retrodiscal tissue (8).

Most of the sensory neurons providing axons to the TMJ have their primary cell bodies within the TG in its mandibular division, as exemplified in various animal models (9–13). In the trigeminal sensory system, the mesencephalic nucleus in the brainstem (MeV) houses a separate population of somatosensory neurons, termed proprioceptors, that collects positional information from the skeletal muscles of the head and neck including those associated with the TMJ. It is not known if there is any consequence of this anatomical separation of proprioceptors from other somatosensory neurons innervating the TMJ.

All trigeminal sensory information passes through the trigeminal nerve root to the trigeminal nuclear complex (TNC) in the midbrain, pons, and medulla of the brainstem. The motor neurons associated with the muscles of mastication associated with CNV3 reside in the TNC in the trigeminal motor nucleus. The motor neuron axons exit the brainstem at the trigeminal motor nerve root, travel with the mandibular nerve and subdivisions, and then directly synapse on muscle cells to trigger voluntary (and reflex) contractions (3). Integration of sensory afferent information with motor efferent information is important in determining whether a motor neuron will be activated to produce a motor output. While the field does not have a full understanding of the complex coordination of the sensory-motor input-output response, it is understood that an interruption in the sensory input at any level of neuron would impact an organism's response (14).

## Trigeminal somatosensory neurons and innervation of the temporomandibular joint

Mammals rely on their senses to provide an authentic representation of the environment that enables appropriate behaviors toward survival. Somatosensation includes the sensory information originating from the skin, limbs, and joints, and encompasses touch, temperature, body position, detection of chemicals, and pain. Recent work demonstrates that the somatosensory system also receives information from many other tissues including the muscles, lymph nodes, teeth, and meninges (15–20) contributing to interoception.

Stimuli that activate somatosensory neurons produce a neuronal electrical impulse that is relayed via axons to the CNS. Somatosensory neurons of the trigeminal system are activated by stimuli from the orofacial cavity, and these impulses then travel through the trigeminal nerve. Recent research has suggested that, rather than just acting as a site of transition from the PNS to the CNS, the trigeminal nerve may modulate signals received from afferent endings prior to these impulses being sent to the ipsilateral TNC (21). Modulation of the signal at this step of the somatosensation process may serve as a target prior to the CNS. Of note, while all somatosensory information projects to the TNC, the discrete targets within the complex depend on the stimulus modality and associated peripheral structures. As previously noted, canonical proprioceptive input arrives to the MeV; innocuous mechanical input arrives to the principal sensory nucleus; and thermal, chemical, and nociceptive input arrives to the spinal trigeminal nucleus (22). How these diverse sensory inputs are coordinated at the level of the TNC or brainstem regions to give rise to TMJ movement remains largely unexplored.

Somatosensory neurons may be defined broadly based on the physiological stimulus on or within tissues that triggers their activation (23): mechanoreceptors, thermoreceptors, chemoreceptors (24), and proprioceptors. Additionally, physical stimuli that produce tissue damage (i.e., noxious) recruit the activation of nociceptors, which are often associated with the subsequent perception of pain (25), and elicit responses to prevent further tissue damage (Table 2). The preceding categorization of somatosensory neurons is highly simplistic considering (1) position and movement of tissue produces mechanical forces indicating that proprioceptors are a subcategory of mechanoreceptors; (2) categories are not mutually exclusive and somatosensory neurons may receive multiple designations; and (3) noxious stimulation is likely to recruit activation of somatosensory neurons that also encode innocuous stimulation (26). To address this in part, additional terminology exists including the term polymodal nociceptor to label a somatosensory neuron that responds to noxious stimuli of multiple modalities.

**Table 2:** Functional classification of sensory receptors (neurons).

Classification	Stimuli detected
Mechanoreceptors	Mechanical deformation, vibration, and pressure applied to tissue
Thermoreceptors	Heating or cooling of tissue
Chemoreceptors	Chemical changes within tissue
Proprioceptors	Musculoskeletal positional change
Nociceptors	Damaging stimuli to tissue (multi-modal)

Other traditional definitions of PNS neurons are based on electrophysiological measurements of axonal conduction velocity as well as histological measures of nerve fiber diameter. From this, three types were outlined: the myelinated Type A and Type B fibers, and the non-myelinated Type C fibers. Type A fibers represent sensory and motor axons and are further subdivided by diameter size and conductance velocities. Type B fibers are preganglionic autonomic axons with smaller diameters and lower conductance velocities than Type A fibers. Type C fibers, which include sensory axons and autonomic postganglionic fibers, are the smallest of the fibers and have the lowest conductance velocities due to the absence of myelination (27) (Table 3).

**Table 3:** Traditional classification of nerve fibers.

Fiber type	Subtype	Diameter (μm)	Conduction velocity (m/s)	Description/Function
A				Myelinated; sensory and motor axons
	*α*	24–40	70–120	Relay proprioception
	*β*	10–24	30–70	Relay touch
	*γ*	6–12	15–30	Relay touch
	*δ*	4–10	12–20	Relay pain and temperature
B		<6	3–15	Myelinated; preganglionic autonomic axons
C		1–4	0.5–2.3	Unmyelinated; sensory axons and autonomic postganglionic axons; relay pain, temperature, touch and itch

Arguably the conduction velocity of a fiber may simply indicate how quickly information will arrive to the brainstem. However, the traditional classification of neurons has been roughly correlated to the function of neuronal subtypes based on the response profile of their fibers. Aβ fibers have been associated with detecting light touch due to their low threshold responses to mechanical forces. The majority of thinly myelinated Aδ and C fibers were thought to carry nociceptive signals based on responses to noxious stimuli (2, 27, 28)*.* Non-myelinated C fibers were further divided based on the type of information that they relay, receiving designations including high-threshold mechanoreceptors, thermoreceptors, chemoreceptors, polymodal nociceptors that respond to both noxious mechanical and temperature, and silent receptors that respond only after the induction of inflammation (25)*.* While this traditional classification of somatosensory neurons provides important physiological information about associated fibers, they offer a limited view of sensory innervation.

As mentioned earlier, the TMJ is innervated posteriorly by the auriculotemporal nerve, anteriorly by the masseteric nerve, and anteromedially by the deep temporal nerve, all of which originate from the CNV3 (5). The somatosensory neurons that contribute to TMJ innervation are nociceptors, proprioceptors, mechanoreceptors, chemoreceptors, and thermoreceptors with nociceptors, proprioceptors, and mechanoreceptors representing the majority of somatosensory innervation. Somatosensory innervation of the TMJ is essential as it aids in the proper position and movement of the joint, as well as the perception of noxious or pain-producing stimuli (29, 30). Sensory neurons innervating the joint, particularly the condylar area, represent mechanoreceptors that respond to jaw movements. The majority of these mechanoreceptors are thought to be slow-adapting and detect jaw opening whereas a smaller percentage are fast-adapting and signal jaw movements outside the typical range of motion (31, 32).

Human cadavers have been used in prior research to elucidate the composition of sensory innervation to distinct structures of the TMJ. Muscles, such as the masseter and temporalis, were shown to be innervated by both myelinated Aδ and unmyelinated C-fiber trigeminal afferents. The afferents feature nonspecialized endings that project to the trigeminal subnucleus interpolaris and caudalis, and relay noxious mechanical and chemical stimuli (33) indicating they are probable nociceptors and/or chemoreceptors. Work to study the density of innervation within the masseter muscles suggested that the upper posterior and upper intermediate portions receive denser innervation from the masseteric nerve (34).

Additional work indicated that free nerve endings are more prevalent in connective tissue surrounding muscle cells (35). In addition to the muscle, the anterolateral and posterior capsule, the disk parenchyma (36), ligament insertions, and synovial tissue (37) were shown to receive sensory innervation.

Nociceptive free nerve endings targeting these tissues—whether myelinated or unmyelinated—were either encapsulated or un-encapsulated. Encapsulated free nerve endings associated with mechanosensitive A-type fibers included Pacinian corpuscles, Ruffini endings, and Golgi tendon organs. Ruffini endings and Pacinian corpuscles were found in the joint capsule, while Golgi tendon organs were found in the associated ligaments (38). When looking at the anterior and posterior bands of the disk, as well as the surrounding synovial transition region of the capsule, Golgi tendon organs were localized to the anterior band, Pacinian corpuscles were localized in the posterior band, and Ruffini endings localized in the middle of the anterior band (36) providing a clear indication that there is mechanosensory specialization among portions of the capsule. In human cadavers, no innervation was identified in the articular disk (37).

The TMJ structures are highly conserved across mammals. The genetic tools available in mouse models, as well as having the lowest maintenance costs among rodents, have become especially attractive for the study design toward reproducibility and understanding of molecular mechanisms in TMJ pathology (39). In mice, molecular markers have been used to distinguish groups of somatosensory neurons as well as nociceptors. In some cases, when molecular receptors confer sensitivity to environmental stimuli, neuronal function has been implicated. Ion channels of the transient receptor potential (Trp) family are involved in thermosensation and chemosensation—Trpm8 detects cool temperatures (40, 41), Trpa1 is activated by chemical irritants (42), and Trpv1 senses noxious heat and is potentiated by inflammation (43). The ion channel Piezo2 is responsible for the mechanosensation necessary for touch and proprioception (44, 45). The mas-related G-coupled protein receptor Mrgpra3 is involved in the detection of chemical itch stimuli. Alternatively, some neuropeptides and receptors serve as markers for neurons that contribute to inflammation, pain, and itch—including calcitonin gene-related peptide (Calca/CGRP), natriuretic peptide B (Nppb) and Mrgprd (46, 47). CGRP in the TG has been associated with sensitization processes that are thought to play a role in headaches and facial pain (48).

The complete transcriptional profiles of sensory neurons in mice have revealed that trigeminal somatosensory neurons fall into roughly 13 classes. Most classes feature prominent expression of one or more molecular receptors associated with a sensory function as described above. Overall, thermoreceptors are likely to reside in the populations representing C1-2 or C7-11 since these feature prominent expression of Trp channels. Mechanoreceptors represent neurons in C3-7 and C13 considering these classes feature strong expression of *S100b* (C4-6)*, Piezo2* (C3-7) and *Mrgprd* (C13). Chemoreceptors constitute the C7-12 populations by virtue of the expression of *Trpv1* (C7-11), *Nppb* (C11), and *Mrgpra3* (C12). Also, the expression of *Scn10a* (Nav1.8) provides a reasonable division between nociceptive (C6-13) and non-nociceptive neurons (C1-5) albeit *Scn10a* expression is also present the non-nociceptive C3 class. While “defining” molecular markers may indicate primary sensitivity for each neuronal population, additional molecular sensors may also play an important role in stimulus detection. For instance, high threshold mechanosensation persists in both C6 and C13 following the loss of *Piezo2* (48) (Table 4). Importantly, molecular markers do not always correspond to distinct transcriptional classes of neurons. While *Trpm8* and *Nppb* mark unique populations of trigeminal somatosensory neurons, *Trpv1* and *Piezo2* are found in multiple classes of neurons with functions that are predicted to be divergent.

**Table 4:** Transcriptomic classification of trigeminal neurons.

Transcriptomic classification (von Buchholtz et al.)	Molecular markers	Functional class	Traditional classification
C1	*Trpm8, Nefh, Synpr*	Thermoreceptors	Aδ
C2	*Trpm8, Trpv1, Synpr*	Thermoreceptors	C
C3	*Cd34, Fxyd2, Tmem45b, Synpr, Piezo2, Scn10a*	Mechanoreceptors	C
C4	*Nefh, S100b, Piezo2*	Mechanoreceptors	Aβ/Aδ
C5	*Nefh, S100b, Fxyd2, Piezo2*	Mechanoreceptors	Aβ/Aδ
C6	*Nefh, S100b, Calca, Piezo2, Scn10a*	Mechanoreceptors, Nociceptor	Aβ/Aδ
C7	*Calca, Trpv1, Tmem233, Piezo2, Scn10a*	Thermoreceptors, Mechanoreceptors, Chemoreceptors, Nociceptor	Aδ
C8	*Calca, Trpv1, Trpa1, Scn10a*	Thermoreceptors, Chemoreceptors, Nociceptor	C
C9	*Calca, Trpv1, Scn10a*	Thermoreceptors, Chemoreceptors, Nociceptor	C
C10	*Calca, Trpv1, Sstr2, Scn10a*	Thermoreceptors, Chemoreceptors, Nociceptor	C
C11	*Fxyd2, Trpv1, Nppb, Etv1, Tmem233, Tmem45b, Scn10a*	Thermoreceptors, Chemoreceptors, Nociceptor	C
C12	*Fxyd2, Calca, Etv1, Tmem233, Tmem45b, Synpr, Mrgpra3, Scn10a*	Chemoreceptors, Nociceptor	C
C13	*Fxyd2, Tmem233, Tmem45b, Synpr, Mrgprd, Scn10a*	Mechanoreceptors, Nociceptor	C

Recent work identified and differentiated “backtraced” trigeminal sensory neurons innervating the masseter. Overall, roughly 56% of neurons were A-type fibers with the remainder representing C-fiber type neurons. Nociceptors that were marked by Nav1.8 (*Scn10a*) constituted both A-type and C-fiber neurons and represented the majority (∼61%) of all sensory neurons identified. The four major classes of trigeminal sensory neurons innervate the masseter muscle likely correlate to transcriptional classes: C4 (∼22%, A- LTMRs), C6 (∼24%, Nav1.8+/CGRP+ A-HTMRs), C8-10 (∼15%, Nav1.8+/CGRP+/Trpv1+ C-fibers), and C13 (∼24%, Nav1.8+/IB4+). CGRP+ peptidergic somatosensory neurons have also been found innervating the joint capsule, surrounding areas of the articular disk, and synovial membrane (49) indicating broad anatomical targets and a potential role in diverse pathologies. The lack of expression of Trpv1 in C6 A-HTMRs suggests that these neurons do not represent the C7 classification (18) (Table 5).

**Table 5:** Molecular masseteric trigeminal classification and function.

Masseteric trigeminal classification (Lindquist et al.)	Probable transcriptomic classification (von Buchholtz et al.)	Markers	Function
S1	C13	Nav1.8, IB4	C-nociceptor, non-peptidergic
S2	C13	Nav1.8, IB4	C-nociceptor, non-peptidergic
S3	C8, C9, C10	Nav1.8, CGRP, Trpv1	C-nociceptor peptidergic
S4	C8, C9, C10	Nav1.8, CGRP, Trpv1	C-nociceptor peptidergic
S5	C7	CGRP, TrkC	
M1	C6	Nav1.8, CGRP	A-High Threshold Mechanoreceptor
M2	C7	CGRP, TrkC	
M3	C4	TrkC	A-Low Threshold Mechanoreceptor
M4	C4	TrkC	A-Low Threshold Mechanoreceptor

Other pharmacological and behavioral studies support the presence of Trpv1+ innervation to the masseter muscle and suggest they contribute to masseter pain in TMD (50–52). A subset of these Trpv1+ neurons may also express Trpa1 (51), but it is unclear if this corresponds to discrete roles in sensation. Furthermore, the independent identification of peptidergic nociceptors innervating the masseter via fast blue stain supports the presence of this population. While these neurons were implicated in complete Freund's adjuvant (CFA) and tendon ligation (TASM) induced masseter pain (49), it is not clear if A-type and/or C fiber peptidergic nociceptors are contributing. The masseter is not the only muscle that is impacted by TMD, requiring further investigation of somatosensory innervation to understand the similarities and differences between the muscles of mastication.

Tracing based approaches in mice have strong potential to enable an understanding of how somatosensory innervation contributes to other non-muscle TMJ structures. Importantly, *in vitro* classification of trigeminal somatosensory neurons can be applied to any neurons that are retrogradely-captured via peripheral tissue injections (15). Furthermore, combining transcriptional classification with methods to evaluate TG neuron activity (48) during naturalistic stimulation of the TMJ will directly connect somatosensory neuron subtype to function. However, methods to improve the targeting precision will be necessary to label and define the innervation of the capsule and surrounding connective tissues. For instance, advanced imaging techniques such as ultrasound (53) or magnetic resonance imaging (54) have enabled the targeting of intracranial structures and could be adopted for extracranial TMJ injections. An understanding of the somatosensory neurons that contribute to sensation in distinct joint tissues as well as supporting structures will provide a basis for understanding how innervation encodes and coordinates jaw movement, and may be altered in models of TMJ disorders (see below).

## Temporomandibular joint disorders and mechanisms of pain

**Figure 2:**
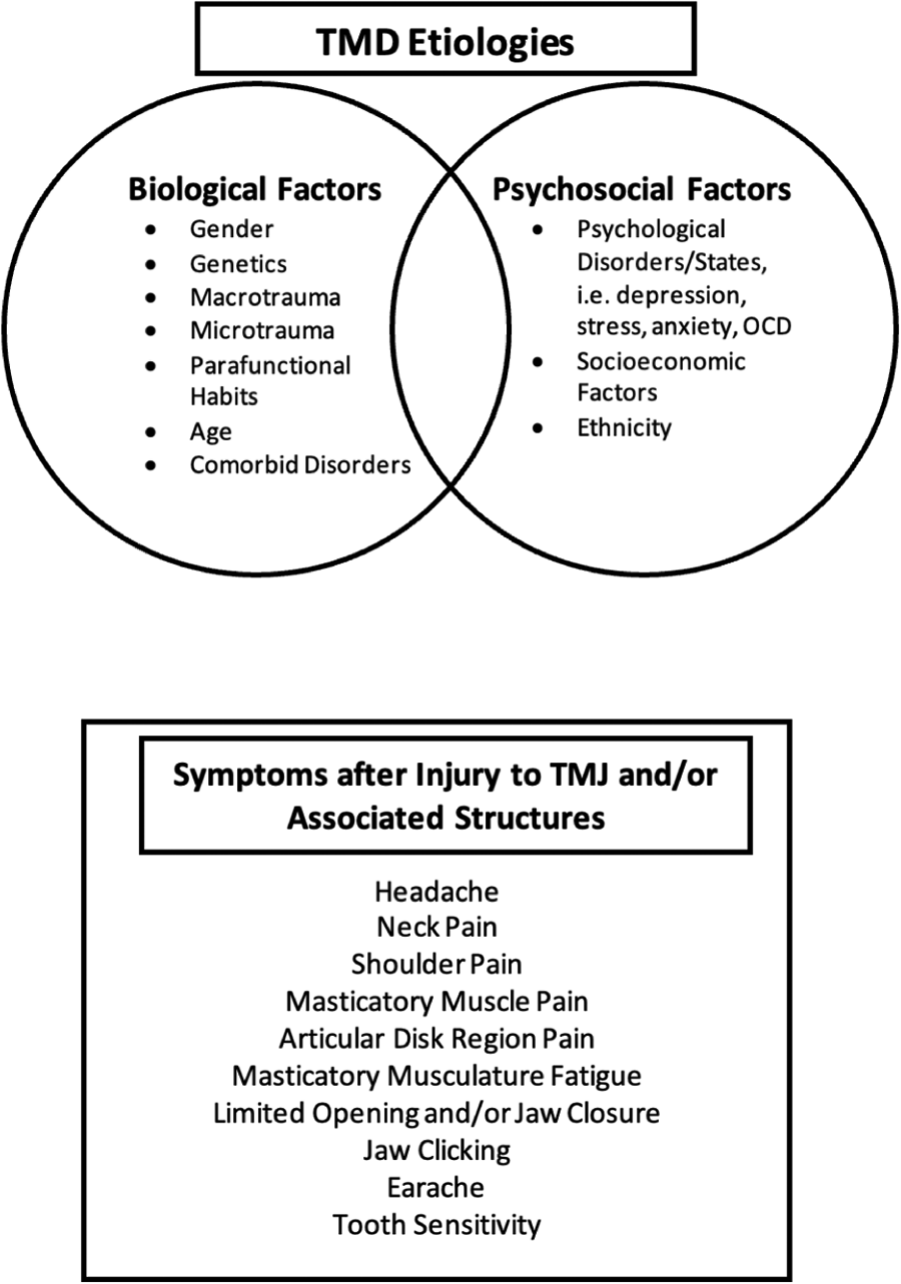
Temporomandibular disorders etiologies and symptoms. Overview of the factors—biological and psychosocial factors—that play a role the development and perpetuation of TMD, and the associated symptoms that patients may experience when injury to one or more TMJ structures occurs.

Temporomandibular joint disorders (TMD) are a group of conditions that cause dysfunction of the TMJ or associated masticatory muscles. TMD arises from diverse etiologies and has varying symptom presentation (Figure 2). While TMD represents one of the most common forms of non-odontogenic craniofacial pain (55) it is not always associated with the perception of pain.

Pain an unpleasant sensory or emotional experience associated with actual or potential tissue damage (56). Pain may be categorized by etiology, arising from a combination of nociceptive, inflammatory, and neuropathic origins. Nociceptive pain is typically preceded by the activation of nociceptive somatosensory neurons (described above) by physical or chemical damage to tissues. The subjective quality of nociceptive pain matches with the noxious stimulus of its origin. Inflammatory pain is a response beyond the initial stimuli stemming from tissue damage. Neuropathic pain is due to injury or disease that damages the nerves or structures associated with the central nervous system (6).

Pain is also classified as either acute or chronic. Acute pain, which typically includes nociceptive and inflammatory pain, is pain initiated from damage (57). The processing of acute pain starts with the activation of these nociceptors by extreme stimuli following either external or internal injury. These initial signals that precede pain are fired by either the fast-conducting Aδ or slow conduction C-fibers—associated with what is generally termed “fast pain” and “slow pain,” respectively (58). These signals are transduced along the axons, and in the case of craniofacial pain, relayed to cell bodies in the TG (as previously mentioned). Incoming information is modulated at the soma prior to continuing to higher order neurons of the CNS in the trigeminal spinal caudalis and thalamus that account for the processing of the perception of trigeminal pain (59). Acute pain abates with the removal of stimuli and/or healing of the injury. Unlike acute pain, chronic pain persists >3 months or beyond what would be defined as normal healing time (60) and interrupts normal tissue function. Thus, chronic pain becomes paradoxical and without benefit.

Both acute and chronic pain are thought to originate with the activation of nociceptors and follow similar ascending and descending pain pathways. While we have a general understanding of processes underlying acute trigeminal pain as described briefly above, the field lacks an understanding of mechanisms contributing to the development of chronic pain. Several models are plausible: (1) Chronic peripheral activation may result in the remodeling of the central circuitry (61) whereby neurons adapt and react more frequently due to lowered thresholds—particularly the lowering of mechanical and heat-sensitive thresholds (62)—thus contributing to the development of chronic pain (63). If constant activation of nociceptors is driving an acute to chronic transition in TMD, we would predict the initiating stimulus would be of mechanical or chemical origin and activate mechano- or chemo-nociceptors innervating the TMJ. (2) When damage to the TMJ and associated muscles triggers inflammatory responses that do not resolve due to continuing injury, peripheral sensitization could lead to central sensitization (64). As previously proposed (65), C-fibers and their characteristic slow synaptic currents coupled with repetitive noxious stimuli may drive the development of central sensitization. (3) If TMJ injury produces nerve damage and/or denervation, the reinnervation process by injured endings or branching of undamaged endings may result in inappropriate activation of nociceptors. Nerve growth factor-mediated expansion of joint capsule innervation has been proposed to underlie TMD pain in osteoarthritis (66). Furthermore, sprouting of sensory neurons has been demonstrated following nerve injury in the skin (67), but has not yet been examined in TMD. Understanding how the sensory innervation is altered in models of TMD using mouse genetic tools would indicate a possible cellular and anatomical origin for peripheral mechanisms that drive the development of chronic pain. Furthermore, determining the activity of sensory innervation in the context of inflammation may indicate the origin of peripheral inputs that drive sensitization and represent a target to prevent the development of chronic pain.

## Current treatment approaches for pain in temporomandibular disorders

Diagnosis of TMD is predominantly based on reviewing medical history and physical examination of the TMJ according to the Diagnostic Criteria for Temporomandibular Disorders (DC/TMD) protocols. From the patient history, signs, and symptoms, the diagnosis of TMD is made in addition to its type and severity (68). Imaging studies, including x-ray, magnetic resonance, or computing tomography support accurate diagnosis of TMD (69).

Treatment of TMD is dependent on type and severity and can involve nonsurgical or surgical approaches (Table 6). The majority of TMD cases are temporary and may or may not be associated with acute pain. Fortunately for most patients, TMD resolves either without intervention or in response to nonsurgical treatments. Initial recommendations are often conservative, aimed at addressing acute pain in the least intrusive, damaging way. Some possible treatments include diet modification to softer foods and warm or cold compress applied to symptomatic muscle(s). Medications may be recommended (or prescribed) to reduce pain and inflammation, or treat any associated psychosocial disorders (discussed above) that may act as initiating or perpetuating factors of TMD, and include nonsteroidal anti-inflammatory drugs (NSAIDs), muscle relaxants, corticosteroids, benzodiazepines, and tricyclic antidepressants (68).

**Table 6:** Treatments for temporomandibular disorders.

Non-surgical treatment	Minimally invasive treatment	Surgical treatment
• Diet modification • Cool or heated compress to muscle • Over-the-counter analgesics • Prescribed analgesics • Prescribed medication for psychosocial disorders • Physical therapy • Intraoral appliances • Dentition modification	• Botulinum toxin injections • Lidocaine injections	• Arthrocentesis • Arthroscopy • TMJ implant

The multifactorial nature of TMD requires the targeting of different pain and symptoms to remedy the disorder. Over-the-counter and prescribed medications are aimed at various targets. Benzodiazepines, tricyclic antidepressants (70), and opioids (71) are examples of drugs that target the central nervous system. Lidocaine, which blocks voltage-gated Na+ channels (72); NSAIDs, which inhibit cyclooxygenase in peripheral tissue (73); opioids, which also have peripheral targets (74); botulinum toxins, which can act at the neuromuscular junction, autonomic ganglia, postganglionic parasympathetic nerve endings, and postganglionic sympathetic nerve endings (75); are all drugs that inhibit the transfer of pain from the periphery to the central nervous system. Physical therapy, including, but not limited to thermal ultrasound, chiropractic therapy, craniomandibular therapy, and exercise rehabilitation (76, 77), have been shown to partially and/or completely remedy symptoms in some forms of TMD excluding systemic or whiplash-related TMD (78, 79). The effectiveness of these peripheral treatments indicates that a portion of TMD cases associated with pain may stem from somatosensory activation (e.g., nociceptors). The precise cellular and/or molecular targets associated with somatosensory neurons are not known.

Intraoral appliances, such as an occlusal splint may also be provided to help improve TMJ function. Of non-surgical treatments, occlusal splints protect the dentition and may stabilize and distribute forces to the TMJ. There is debate concerning the effectiveness of occlusal splints in treating TMD. Some note that splints are unlikely to prevent the continuation of clenching or bruxism, and/or eliminate associated pain (80). In contrast, considering irregular occlusal contact may lead to imbalanced applied pressure on muscles of mastication, occlusal splints have been reported to provide equal distribution of pressure on the jaw, reduce pain intensity in acute and chronic conditions, and improve movement capabilities of the jaw (81), as well as decrease experienced pain from headaches and disc displacements in some patients (82). Overall, modification of the dentition to achieve balanced occlusal forces across the dentition, including crown placement, occlusal surface reduction, or orthodontic treatments (69) may only have limited benefit.

In cases of severe acute or chronic pain resulting from TMD, clinicians will opt for minimally-invasive or surgical interventions. Minimally-invasive treatments, while not the first route of suggested treatment, tend to be preferred to invasive surgical interventions for TMD. Direct injection of botulinum toxin (BTx), a muscle relaxant, into muscles is frequently used to reduce myofascial pain in TMD (80). It acts on the ascending pain pathway, blocking the release of acetylcholine from the presynaptic terminal end at the neuromuscular joint thereby preventing muscle contractions (83). It should be noted that the therapeutic effect of BTx injection is reversible and requires repeated injections for long-term treatment a continued effect, which thus is not a preferred method of long-term treatment for patients. Importantly, BTx injection is ineffective for treating cartilaginous degenerative TMD, hinting at a differential somatosensory origin for pain in the TMJ compared to the muscle. Of note, while lidocaine, an amide local anesthetic, can also be injected intramuscularly to relieve pain, it has shown less efficacy. Similar to BTx, lidocaine reduces sensation only temporarily, thus injections must be administered as frequently as once a month (84). The minimal benefit that has been demonstrated with lidocaine injection may underscore the uneven distribution of muscle innervation by sensory neurons that initiate myofascial pain.

When non- and minimally-invasive therapies fail, invasive surgeries are the last resort for arthralgias. Unfortunately, no additional options are available for myalgias. Arthrocentesis and arthroscopy are procedures to access and manipulate the TMJ. When scar-like tissues have developed because of degeneration of the disk, arthrocentesis—injecting liquid to flush out the scar tissue—or arthroscopy—the insertion of an instrument to directly remove scar tissue—can be conducted. These procedures have been shown to be comparable to the conservative methods of TMD treatments mentioned above, able to alleviate TMD associated pain and increase functionality of the joint in some patients (85). An alternative to arthrocentesis and arthroscopy has been the injection of platelet rich plasma or platelet rich fibrin into the joint, with clinical results showing reduced pain symptoms and joint function improvement (86–88). Lastly, a more permanent alteration of the joint can be conducted via open surgery: TMJ implants can be inserted to replace a part or the entirety of the joint. Surgical treatments are an involved process that are not guaranteed to alleviate TMD and/or associated pain (69). Currently, the treatments for chronic TMD are temporary in relief (89). This is of major concern, as TMD impacts all aspects of life, underscoring the need for new treatments. We propose that a better understanding of how somatosensory innervation is altered during the development and progression of TMD will lead to the ability to target is distinct etiologies of pain.

## Conclusion

In this review, we emphasize the need to further investigate the somatosensory innervation of the TMJ toward understanding the development of TMD pain. We briefly discuss how the unique movements of the mammalian mandible are due to unique aspects of the anatomy of the TMJ. We then provide an overview of the innervation of the TMJ and associated masticatory muscles by the trigeminal nerve (CNV) emphasizing the somatic and sensory components of the system. We outline subtypes of somatosensory neurons, their molecular differentiation, and their contribution to innervation of the human and mouse TMJ. We give an overview of the etiologies and symptoms of disorders of the TMJ (TMD) and propose possible mechanisms contributing to chronic pain in TMD. Lastly, we discuss current treatments available to address the dysfunction and pain caused by TMD.

Nociceptors contribute to the perception of pain, yet we lack an understanding of how the components of the TMJ are innervated by this subtype of somatosensory neurons. Future studies using high-resolution imaging may offer a strategy to target the different structures that make up the TMJ and masticatory muscles. Furthermore, genetic mouse lines, transcriptomic classification, and functional imaging of TG neurons will outline the contribution of nociceptors to TMJ structures, their physiological function, as well as their modification with the development of TMD. Ultimately, these studies would provide strategies for targeting somatosensory neurons, in particular nociceptors, for more effective treatments for chronic pain experienced with TMD.

## Group members of RE-JOIN Consortium

“The RE-JOIN Consortium includes Taeyoung Ahn, Kyle Allen, Alejandro Almarza, Benjamin Arenkiel, Maryam Aslam, Basak Ayaz, Yangjin Bae, Bruna Balbino de Paula, Mario Boada, Dawen Cai, Robert Caudle, Racel Cela, Rui Chen, Yong Chen, Brian Constantinescu, Yenisel Cruz-Almeida, Chris Donnelly, Zelong Dou, Joshua Emrick, Malin Ernberg, Spencer Fullam, Janak Gaire, Akash Gandhi, Terese Geraghty, Benjamin Goolsby, Stacey Greene, Nele Haelterman, Jung-Chien Hsieh, Zhiguang Huo, Michael Iadarola, Shingo Ishihara, Sudhish Jayachandran, Zixue Jin, Alisa Johnson, Frank Ko, Priya Kulkarni, Zhao Lai, Brendan Lee, Yona Levites, Jun Li, Martin Lotz, Tristan Maerz, Anne-Marie Malfait, Maryann Martone, Maggie McGlothlin, Lindsey McPherson, Bella Mehta, Rachel Miller, Richard Miller, Mike Newton, Alia Obeidat, Soo Oh, Merissa Olmer, Dana Orange, Miguel Otero, Kevin Otto, Folly Patterson, Danial Perez, Sienna Perry, Ted Price, Russell Ray, Dongjun Ren, Margarete Ribeiro Dasilva, Alexus Roberts, Elizabeth Ronan, Oscar Ruiz, Shad Smith, Mariobys Soccorro Gonzalez, Kaitlin Southern, Josh Stover, Michael Strinden, Hannah Swahn, Evelyn Tantry, Cristal Villalba Silva, Robin Vroman, Joost Wagenaar, Lai Wang, Kim Worley, Joshua Wythe, Jiansen Yan, Julia Younis.”
