# Corrigendum: Trigeminal somatosensation in the temporomandibular joint and associated disorders

**DOI:** 10.3389/fpain.2024.1454278

**Published:** 2024-07-05

**Authors:** Sienna K. Perry, Joshua J. Emrick

**Affiliations:** Department of Biologic and Materials Sciences & Prosthodontics, School of Dentistry, University of Michigan, Ann Arbor, MI, United States

**Keywords:** orofacial innervation, TMJ, TMD, trigeminal, somatosensation, chronic pain, orofacial pain, nociceptors

A Corrigendum on Trigeminal somatosensation in the temporomandibular joint and associated disorders By Perry SK, Emrick JJ, for RE-JOIN Consortium (2024). Front. Pain Res. 5:1374929. doi: 10.3389/fpain.2024.1374929

**Text Correction**

In the published article, there was an error. Two names of group members of RE-JOIN Consortium were misspelled (Cristal Villalba Silva and Anne-Marie Malfait) and two names of group members of RE-JOIN Consortium were omitted (Jung-Chien Hsieh and Maggie McGlothlin).

A correction has been made to Group members of RE-JOIN Consortium. The section previously stated:

“The RE-JOIN Consortium includes Taeyoung Ahn, Kyle Allen, Alejandro Almarza, Benjamin Arenkiel, Maryam Aslam, Basak Ayaz, Yangjin Bae, Bruna Balbino de Paula, Mario Boada, Dawen Cai, Robert Caudle, Racel Cela, Rui Chen, Yong Chen, Brian Constantinescu, Yenisel Cruz-Almeida,Chris Donnelly, Zelong Dou, Joshua Emrick, Malin Ernberg, Spencer Fullam, Janak Gaire, Akash Gandhi, Terese Geraghty, Benjamin Goolsby, Stacey Greene, Nele Haelterman, Zhiguang Huo, Michael Iadarola, Shingo Ishihara, Sudhish Jayachandran, Zixue Jin, Alisa Johnson, Frank Ko, Priya Kulkarni, Zhao Lai, Brendan Lee, Yona Levites, Jun Li, Martin Lotz, Tristan Maerz, Anne-Marier Malfait, Maryann Martone, Lindsey McPherson, Bella Mehta, Rachel Miller, Richard Miller, Mike Newton, Alia Obeidat, Soo Oh, Merissa Olmer, Dana Orange, Miguel Otero, Kevin Otto, Folly Patterson, Danial Perez, Sienna Perry, Ted Price, Russell Ray, Dongjun Ren, Margarete Ribeiro Dasilva, Alexus Roberts, Elizabeth Ronan, Oscar Ruiz, Shad Smith, Mariobys Soccorro Gonzalez, Kaitlin Southern, Josh Stover, Michael Strinden, Hannah Swahn, Evelyn Tantry, Cristal Villalna Silva, Robin Vroman, Joost Wagenaar, Lai Wang, Kim Worley, Joshua Wythe, Jiansen Yan, Julia Younis.”

The corrected sentence appears below:

“The RE-JOIN Consortium includes Taeyoung Ahn, Kyle Allen, Alejandro Almarza, Benjamin Arenkiel, Maryam Aslam, Basak Ayaz, Yangjin Bae, Bruna Balbino de Paula, Mario Boada, Dawen Cai, Robert Caudle, Racel Cela, Rui Chen, Yong Chen, Brian Constantinescu, Yenisel Cruz-Almeida, Chris Donnelly, Zelong Dou, Joshua Emrick, Malin Ernberg, Spencer Fullam, Janak Gaire, Akash Gandhi, Terese Geraghty, Benjamin Goolsby, Stacey Greene, Nele Haelterman, Jung-Chien Hsieh, Zhiguang Huo, Michael Iadarola, Shingo Ishihara, Sudhish Jayachandran, Zixue Jin, Alisa Johnson, Frank Ko, Priya Kulkarni, Zhao Lai, Brendan Lee, Yona Levites, Jun Li, Martin Lotz, Tristan Maerz, Anne-Marie Malfait, Maryann Martone, Maggie McGlothlin, Lindsey McPherson, Bella Mehta, Rachel Miller, Richard Miller, Mike Newton, Alia Obeidat, Soo Oh, Merissa Olmer, Dana Orange, Miguel Otero, Kevin Otto, Folly Patterson, Danial Perez, Sienna Perry, Ted Price, Russell Ray, Dongjun Ren, Margarete Ribeiro Dasilva, Alexus Roberts, Elizabeth Ronan, Oscar Ruiz, Shad Smith, Mariobys Soccorro Gonzalez, Kaitlin Southern, Josh Stover, Michael Strinden, Hannah Swahn, Evelyn Tantry, Cristal Villalba Silva, Robin Vroman, Joost Wagenaar, Lai Wang, Kim Worley, Joshua Wythe, Jiansen Yan, Julia Younis.”

The authors apologize for this error and state that this does not change the scientific conclusions of the article in any way. The original article has been updated.

